# Frailty syndrome and rehospitalizations in elderly heart failure patients

**DOI:** 10.1007/s40520-017-0824-6

**Published:** 2017-08-28

**Authors:** Izabella Uchmanowicz, Maria Kuśnierz, Marta Wleklik, Beata Jankowska-Polańska, Joanna Jaroch, Krystyna Łoboz-Grudzień

**Affiliations:** 1 Division of Nursing in Internal Medicine Procedures, Department of Clinical Nursing, Faculty of Health Sciences, Wroclaw Medical University, 5 Bartla Street, 51-618 Wroclaw, Poland; 2 Wrocław Department of Cardiology, T. Marciniak Lower Silesian Hospital, 2 Fieldorfa Street, 54-049 Wroclaw, Poland

**Keywords:** Frailty syndrome, Aging, Rehospitalizations, Heart failure

## Abstract

**Background:**

Heart failure (HF) patients with frailty syndrome (FS) are at higher risk of falling, decreased mobility, ability to perform the basic activities of daily living, frequent hospitalizations, and death.

**Aims:**

The purpose of this study was to evaluate the correlations between FS and hospital readmissions, and to assess which factors are associated with rehospitalizations.

**Methods:**

The study included 330 patients with a mean age of 72.1 ± 7.9 years, diagnosed with HF. Frailty was measured using the Polish version of the Tilburg Frailty Indicator (TFI). Demographic, sociodemographic, and clinical data, such as the New York Heart Association (NYHA) functional class, ejection fraction (EF), number of rehospitalizations, and the medications taken, were obtained.

**Results:**

Positive correlation was observed between the number of hospitalizations and FS. In the single-factor correlation analysis, treatment with diuretics, a higher NYHA class, and a lower left ventricular EF were predictors of a higher number of hospitalizations. Additionally, the physical and psychological components of the TFI, as well as the total TFI score, predisposed HF patients to more frequent hospitalizations.

**Discussion:**

It seems that a deterioration of functional capabilities and an increase in symptom severity naturally lead to increased hospitalization frequency in HF. In the own study, regression analysis indicates that high NYHA classes and TFI social component scores are significant predictors of the number of hospitalizations in the studied group.

**Conclusions:**

FS is highly prevalent among elderly HF patients. Higher frailty levels in elderly patients are a determinant of more frequent rehospitalizations in HF.

## Background

Heart failure (HF) is the most common cause of hospitalization for patients older than 65. Despite developments in cardiovascular treatment, the high hospitalization rate has not changed for the last 20 years, and is currently one of the most significant challenges for health care systems worldwide [1]. Epidemiological data show that following a first hospitalization due to HF, 25% of patients are rehospitalized within 30 days, and 50% are rehospitalized within 6 months of the first hospitalization [2]. Within 5 years of diagnosis, 43% of HF patients are hospitalized five or more times [3]. HF is an increasingly serious epidemiological and clinical issue—the numbers of patients with HF are growing, due to factors including longer lifespans and higher survival rates of patients with acute coronary syndrome.

Given its clinical course, HF puts elderly patients in repeated situations of stress and vulnerability. Therefore, frailty syndrome (FS) may be particularly relevant in HF patients. FS is one of the key issues in present-day geriatrics and an exponent of old biological age. Patients diagnosed with FS are at higher risk of falling, decreased mobility, decreased ability to perform the basic activities of daily living, frequent hospitalizations, and death [4]. Studies show that 25–50% of cardiovascular patients suffer from FS. Patients with HF and concurrent FS are at a higher risk of experiencing the adverse effects of their disease, compared with non-frail patients [5, 6]. The prevalence of FS is currently approx. 40% and epidemiological forecasts indicate that it will rise as the population ages [6, 7]. Identifying FS in patients with HF is clinically significant, as the syndrome adversely affects the patients’ prognosis. FS in patients with HF increases the 1-year mortality rate [7, 8], and may significantly complicate the process of diagnosing HF [8, 9].

Frailty is internationally recognized as an important medical syndrome and is defined as an age-associated biological condition characterized by decreased biological reserves due to dysregulation of several physiological systems [10]. Currently, there are two approaches to FS. The first is based on the definition based on the FS phenotype proposed by Fried et al. [11]. The second derives from the deficit index, which allows us to determine the severity of FS through verification of existing bio-psycho-social deficits [12], and the accumulation of deficits, i.e., age-associated health disorders (symptoms, physical features, diseases, disability, and abnormal laboratory test results). A high Frailty Index was identified as a predictor of mortality in a population observed for 5 years [12].

The most recent definitions of FS adopt a multidimensional view: frailty is defined as a dynamic state affecting an individual who experiences losses in one or more domains of human functioning (physical, psychological, and social), which is caused by the influence of a range of variables and which increases the risk of adverse outcomes [11]. The present study is based on a multidimensional definition of frailty.

The assessment of frailty in HF elderly patients during their hospitalization may be challenging because we do not know to what extent the underlying mechanism of FS in these patients is related to their biological condition, including functional impairment or the presence of comorbidities. However, the identification of FS in patients with HF may be important from the clinical point of view, as this condition adversely affects the course of the disease and in addition may also have an impact on hospital readmissions.

## Aim

Considering the increasing age of HF patients, a special approach to their treatment is required, with more attention paid to geriatric conditions, e.g., FS. The purpose of this study was to evaluate the correlations between FS and hospital readmissions, and to assess which factors are associated with rehospitalizations.

## Methods

### Study design and sample

The study was performed in the Cardiology Ward in Wrocław, Poland. Data were collected from January 2015 through September 2015. It included 330 patients (220 men, 148 women) with a mean age of 72.1 years (±7.9 years), diagnosed with HF. The inclusion criteria were as follows: (1) clinically confirmed HF diagnosis, (2) the patient’s written informed consent, and (3) age ≥60 years. The exclusion criteria were as follows: (1) with moderate to severe dementia (defined as a Mini Mental Score ≤15) [11], (2) requirement for intensive cardiac care, and (3) previous stroke.

### Ethical consideration

The study was approved by the Bioethics Committee of the Wrocław Medical University, approval no. KB 521/2014.

### Measurement tools

Demographic and sociodemographic data (age, gender, years of education, marital status) were obtained from interviews performed by a cardiac nurse and patient records. Clinical data such as the New York Heart Association (NYHA) functional class, ejection fraction (EF), number of rehospitalizations due to HF during 1 year, and the medications taken were obtained from medical records and personal interviews with the participants performed by a cardiac nurse.

Frailty was measured using the Polish version of the Tilburg Frailty Indicator (TFI) [13, 14]. The TFI consists of two parts. One addresses the sociodemographic characteristics of a participant (gender, age, marital status, country of origin, educational level, and monthly income) and other potential determinants of frailty (lifestyle, multimorbidity, life events, and home living environment). The second part addresses components of frailty. Part two of the TFI comprises 15 self-reported questions, divided into three domains. The physical domain (0–8 points) consists of eight questions related to physical health, unexplained weight loss, difficulty in walking, balance, hearing problems, vision problems, strength in hands, and physical tiredness. The psychological domain (0–4 points) comprises four items related to cognition, depressive symptoms, anxiety, and coping. The social domain (0–3 points) comprises three questions related to living alone, social relations, and social support. Eleven items of part two of the TFI have two response categories (“yes” and “no”), while the remaining items have three (“yes”, “no,” and “sometimes”). “Yes” or “sometimes” responses are scored 1 point each, while “no” responses are scored 0. The instrument’s total score may range from 0 to 15; the higher the score, the higher the patient’s frailty. Frailty is diagnosed when the total TFI score is ≥5. Previous studies suggest that the TFI is a valid and reliable instrument for measuring frailty [9, 13–18].

### Statistical analysis

Participant characteristics were calculated based on frailty and sociodemographic and clinical factors on the number of hospital readmissions a year. The patients were divided into two groups, according to the number of their hospitalizations. The minimal sample size providing 0.5 statistical power of multivariate analysis was estimated at 330 patients. Normal distribution of the TFI scores were verified with the Shapiro–Wilk test and their statistical characteristics were presented as arithmetic means and their standard deviations (SDs), or medians and interquartile ranges (IQRs). Sociodemographic and clinical characteristics were compared with the Kruskal–Wallis test and Dunn’s post hoc test. The power and direction of associations between the level of frailty score and the number of hospital readmissions were determined on the basis of Spearman’s coefficients of rank correlation (*r*_*S*_). The variables that were identified as significant determinants of TFI scores on univariate analysis were included in the multiple regression. Nominal variables (qualitative) in the form of number (*n*) and proportion (%) were included in cross tables; their independence was verified with the Pearson’s Chi-squared test.

## Results

### Sociodemographic and clinical characteristics of the study sample

Statistical analysis was performed on the results of clinical examinations of 330 patients, including 148 women (44.8%), aged 60–94 (mean 72.1 ± 7.9). The patients were divided into two groups, based on the number of hospitalizations: group 1—infrequent hospitalizations (up to 2 a year) and group 2—frequent hospitalizations (3 or more a year). Patients in group 2 (frequent hospitalizations) typically had a higher NYHA functional class—class IV for 49.5% of patients vs 36.5% in group 1 (*p* < 0.001). Patients in group 2 had lower left ventricular EFs: 37.7 vs 40.5% in group 1 (*p* = 0.002). Furthermore, FS co-occurred with HF in 64.8% of patients overall, but in group 2 (frequent hospitalizations) the rate was 71.1%, while in group 1 (up to 2 hospitalizations a year) it was 62.2%.

The basic descriptive statistics for participants in each subgroup (based on the number of hospitalizations) are shown in Table 1.

**Table 1:** The basic descriptive statistics for the studied patients

Characteristic	Total (*n* = 330)	Group 1 (*n* = 233)	Group 2 (*n* = 97)	1 vs 2 *p*
Age (years)				0.312a
M ± SD	72.1 ± 7.9	72.0 ± 7.5	72.4 ± 8.9	
Me (Q1; Q3)	69 (66; 77)	69 (66; 77)	71 (66; 77)	
Female	148 (44.9%)	102 (43.8%)	46 (47.4%)	
Comorbidities				
Diabetes mellitus	159 (48.2%)	117 (50.2%)	42 (43.3%)	0.306b
Hypertension	264 (80.0%)	189 (81.1%)	75 (77.3%)	0.526b
COPD	90 (27,3%)	61 (26.2%)	28 (28.9%)	0.680b
Renal insufficiency	53 (16.1%)	30 (12.9%)	20 (20.6%)	0.255b
NYHA class				< 0.001c
I	19 (5.8%)	19 (8.2%)	0 (0.0%)	
II	160 (48.5%)	121 (51.9%)	39 (40.2%)	
III	133 (40.3%)	85 (36.5%)	48 (49.5%)	
IV	18 (5.4%)	8 (3.4%)	10 (10.3)	
Left ventricular ejection fraction (EF) (%)				0.002a
M ± SD	39.7 ± 9.4	40.5 ± 8.9	37.7 ± 10.5	
Me (Q1; Q3)	40 (35; 45)	42 (35; 46)	35 (30; 45)	
Min ÷ Max	15 ÷ 70	15 ÷ 65	18 ÷ 70	
Frailty syndrome (TFI)	214 (64.8%)	145 (62.2%)	69 (71.1%)	0.157b
TFI physical domain				0.102a
M ± SD	3.9 ± 2.0	3.8 ± 2.0	4.2 ± 2.0	
Me (Q1; Q3)	4 (2; 5)	4 (2; 5)	4 (3; 5)	
Min ÷ Max	0 ÷ 8	0 ÷ 8	1 ÷ 8	
TFI psychological domain				0.436a
M ± SD	1.7 ± 1.0	1.7 ± 1.0	1.8 ± 1.0	
Me (Q1; Q3)	2 (1; 2)	2 (1; 2)	2 (1; 3)	
Min ÷ Max	0 ÷ 4	0 ÷ 4	0 ÷ 4	
TFI social domain				<0.001a
M ± SD	1.3 ± 0.9	1.2 ± 0.9	1.5 ± 0.9	
Me (Q1; Q3)	1 (1; 2)	1 (1; 2)	1 (1; 2)	
Min ÷ Max	0 ÷ 5	0 ÷ 3	0 ÷ 5	

*M* mean, *SD* standard deviation, *Me* median, *Q*1 lower quartile (25th percentile), *Q*3 upper quartile (75th percentile), *Min* lowest value, *Max* highest value, *a* Mann–Whitney *U* test, *b* Yates’ Chi-squared test, *c* Pearson’s Chi-squared test, *TFI* Tilburg Frailty Indicator, *NYHA* New York Heart Association Functional classification, *EF* ejection fraction, *COPD* chronic obstructive pulmonary disease

### Results from the single-factor logistic regression analysis

In the studied group, a weak (*r*_S_ = +0.181) but significant positive correlation was observed between the number of hospitalizations and frailty (*p* < 0.05).

Single-factor analysis was performed for sociodemographic variables (age, sex), clinical variables (NYHA class, left ventricular EF, medication), and TFI domains (physical, psychological, and social) (Table 2).

**Table 2:** Single-factor logistic regression analysis for hospitalization frequency and the variables analyzed

Variable	Rank correlation analysis		Logistic regression	
	*r*_S_	*p*	*b*	*p*
Age	+0.056	0.312	+0.007	0.634
Female	+0.033	0.545	+0.147	0.544
DM	−0.063	0.253	−0.278	0.253
HT	−0.043	0.433	−0.231	0.433
Diuretics	+0.106	0.054	+**0.660**	**0.057**
Beta-adrenolytics	+0.082	0.138	+0.642	0.143
ACE inhibitors	+0.075	0.174	+0.342	0.175
Statins	+0.050	0.362	+0.224	0.362
Antithrombotics	−0.003	0.961	−0.012	0.961
NYHA class	+**0.220**	<**0.001**	+**0.769**	<**0.001**
Left ventricular EF	**−0.173**	**0.002**	**−0.031**	**0.018**
TFI	+**0.144**	**0.009**	+**0.097**	**0.016**
Physical components	+0.090	0.102	+**0.104**	**0.088**
Psychological components	+0.043	0.436	+0.078	0.506
Social components	+**0.194**	<**0.001**	+**0.447**	<**0.001**

*r*_*s*_ Spearman’s rank correlation coefficient, *b* logistic regression coefficient, *p* regression coefficient significance level, *HT* hypertension, *DM* diabetes mellitus, *ACE* angiotensin-converting enzyme, *NYHA* New York Heart Association Functional classification, *EF* ejection fraction, *TFI* Tilburg Frailty Indicator

In the single-factor correlation analysis, treatment with diuretics, a higher NYHA class, and a lower left ventricular EF were predictors of frequent hospitalizations. Additionally, the physical and psychological components of the TFI, as well as the total TFI score, predisposed HF patients to more frequent hospitalizations.

### Results from the multi-factor logistic regression analysis

Subsequently, multiple-factor logistic regression analysis was performed, which included the following variables: NYHA class, left ventricular EF, diuretic treatment, and physical and psychological TFI components (Table 3).

**Table 3:** Multiple-factor logistic regression analysis for hospitalization frequency and the variables analyzed

Variable	*b*	SE_b_	*p*	OR	95% CI
Constant	**−2.80**	**–**	**–**	**–**	**–**
NYHA class	**0.581**	**0.227**	**0.011**	**1.79**	**1.15–2.79**
TFI social domain	**0.527**	**0.222**	**0.018**	**1.69**	**1.10–2.62**
Diuretics	0.460	0.365	0.207	1.58	0.77–3.24
EF	−0.011	0.016	0.498	0.99	0.96–1.02
TFI	−0.096	0.141	0.496	0.91	0.69–1.20
TFI physical domain	0.117	0.185	0.526	1.12	0.78–1.61

Logit frequent hospitalizations = − 2.80 +[0.581 × NYHA] + [0.527 × social components]

*b* logistic regression coefficient, *p* regression coefficient significance level, *SE* standard error, *OR* odds ratio, *CI* confidence interval, *TFI* Tilburg Frailty Indicator, *NYHA* New York Heart Association Functional classification, *EF* ejection fraction

A higher score in the TFI social domain and a higher NYHA class can predict frequent hospitalizations for a patient.

## Discussion

HF is the most common cause of hospitalization for patients older than 65. Despite the developments in cardiovascular treatment, the high hospitalization rate has not changed for the last 20 years. The purpose of this study was to evaluate the impact of FS and hospital readmissions, and to assess variables associated with rehospitalizations.

In the authors’ own study, FS co-occurred with HF in 64.8% of patients overall, but in group 2 (frequent hospitalizations) the rate was 71.1%, while in group 1 (up to 2 hospitalizations a year) it was 62.2%. A similar percentage was reported in the FRAIL–HF study (70.2%) [19], and in the study by McNallan et al. [20] (74%). In the studies quoted, FS was assessed based on the Cardiovascular Health Study definition, while the present study used a Polish adaptation of the TFI. It should be emphasized that international research associations’ consensus recommends both instruments, both for frailty assessment and for frailty screening in elderly patients. In single-factor analysis, FS proved to be a significant predictor for frequent hospitalizations. In the study by McNallan et al. [20], patients with FS had a 65% higher hospitalization frequency than non-frail patients in the 2-year follow-up period. Meanwhile, multiple-factor analysis in the study by Lupon et al. [6] showed frailty to be an independent predictor of mortality, and not of hospitalization frequency, in HF.

Frailty is considered “secondary” or “clinical” when it is associated with known comorbidity and/or disability. Cacciatore et al. [21] reported that diabetes predicts long-term mortality in elderly patients, as well as, clinical frailty significantly predicts mortality in subjects without and even more in those with diabetes, especially in men. The authors concluded that clinical frailty may be considered a new prognostic factor to identify patients with diabetes at high risk of mortality. In addition, Galizia et al. [22] examined the predictive role of frailty on long-term mortality in elderly subjects with and without chronic obstructive pulmonary disease (COPD) and they concluded that clinical frailty may be considered a new prognostic factor to identify COPD subjects at high risk of mortality.

HF is characterized by a frequent instability and until now it is not clear what are the factors determining the rehospitalization of HF-decompensated patients. Testa et al. [23] prospectively determined differences between younger and older patients with decompensated HF. It was observed that older adults had greater comorbidity and disability than younger, were more likely to be hypertensive, and had a more severe clinical profile. Older adults with HF were significantly more likely to receive digitalis, oral anticoagulants, and diuretics and less likely to receive beta-blockers than younger. Finally, they confirmed atrial fibrillation or flutter and HF with preserved EF increased proportionally with increasing age.

It is worth noting that in our study, the social frailty components (questions related to living alone, social relations, and social support) were associated with the hospital readmissions in patients with HF. This finding emphasizes the importance of a multidimensional assessment of frailty, as it means that patients lacking social support were rehospitalized more frequently [24].

Dunbar et al. [25] confirm better adherence and compliance, fewer rehospitalizations, and fewer depressive symptoms found for patients supported by their families and friends. Conroy et al. [26] state that FS is a social issue, requiring efforts to support those living alone and at risk of social isolation. Another study showed a correlation between the social domain of FS, and the self-care and self-control capabilities of patients. Those enjoying social support maintained a proper diet, performed regular physical activity, complied with medication, and were able to recognize symptoms of decompensation [27].

In the own study, an increase in rehospitalization frequency was also observed with higher NYHA functional classes. Mavrea et al. [28] studied the impact of NYHA classes on rehospitalization and reported that NYHA class IV was an independent predictor of rehospitalization in HF patients under 65 years old with preserved EF. The present study also showed that patients who were hospitalized more frequently had a higher NYHA class and a lower EF (<40). In other studies, patients with HF and FS were classified in the NYHA class indicating the high clinical severity of the condition [25].

Therefore, it seems that a deterioration of functional capabilities and an increase in symptom severity naturally lead to increased hospitalization frequency in HF. In the own study, regression analysis indicates that high NYHA classes and TFI social component scores are significant predictors of the number of hospitalizations in the studied group. A higher score in the TFI social domain and a higher NYHA class can predict frequent hospitalizations for a patient. A systematic identification of rehospitalization determinants, including frailty, is important for risk stratification and for introducing the multidisciplinary actions required to prevent symptoms of frailty.

### Implications for practice

From the clinical standpoint, frailty should be assessed to ensure optimum monitoring for patients with chronic HF and to enable the introduction of necessary changes in the therapeutic process. Early identification of FS risk factors is likely to improve the results of HF treatment. Preventing frailty can also contribute to a reduced number of hospitalizations and to a higher quality of life of HF patients.

### Study limitations

The study has some potential limitations. One may be due to the fact that material was collected from HF patients hospitalized in the Cardiology Ward during an exacerbation, which may have influenced results due to the hospitalization, deterioration of health, and symptoms interfering with everyday functioning, experienced at the time.

## Conclusions

Frailty is highly prevalent among elderly HF patients. Higher frailty levels in elderly patients are a determinant of frequent rehospitalizations in HF. For elderly patients with higher scores in the social TFI component, which denotes limited social support, the risk of rehospitalization is higher. Rehospitalization frequency is also significantly affected by a high NYHA class.
